# Supplementary material for: The lived experiences and caring needs of women diagnosed with cervical cancer: A qualitative study in Dar es Salaam, Tanzania
Source: PLoS One. 2023 Aug 10;18(8):e0289925. doi: 10.1371/journal.pone.0289925 (PMC10414621; doi:10.1371/journal.pone.0289925)
Supplement: S1 Text — (DOCX) [file pone.0289925.s001.docx]

**A guide of in-depth interview for the lived experiences and caring needs of cervical cancer patients**

1. How was your initial reaction when you were notified about the diagnosis of the disease?
2. Who did you choose to speak with and what did you discuss when you needed support?
3. How do you see your life after the diagnosis? Please explain in detail about this
4. Can you explain how the family members/relatives handled/interacted with you after the diagnosis of the disease?
5. How was your relationship with your partner after revealing the diagnosis as cervical cancer?
6. Do you think the society has the same attitude towards you after revealing your diagnosis from disease as before?
7. How has been your experience with the healthcare services you receive as far as your illness status is concerned?
8. Is there anything more concerning your experience with the disease condition you would like to add?
