# Supplementary material for: The lived experiences and caring needs of women diagnosed with cervical cancer: A qualitative study in Dar es Salaam, Tanzania
Source: PLoS One. 2023 Aug 10;18(8):e0289925. doi: 10.1371/journal.pone.0289925 (PMC10414621; doi:10.1371/journal.pone.0289925)
Supplement: S2 Text — (DOCX) [file pone.0289925.s002.docx]

# STROBE Checklist- PONE-D-23-10682 (The lived experiences and caring needs of women diagnosed with cervical cancer: A qualitative study in Dar es Salaam, Tanzania).

|  | Item No. | Recommendation | Page  No. | Relevant text from manuscript |
| --- | --- | --- | --- | --- |
| **Title and abstract** | 1 | (*a*) Indicate the study’s design with a commonly used term in the title or the abstract | 1 | The lived experiences and caring needs of women diagnosed with cervical cancer: A qualitative study in Dar es Salaam, Tanzania |
|  |  | (*b*) Provide in the abstract an informative and balanced summary of what was done and what was found | 2-3 | A qualitative descriptive study was carried out among cervical cancer patients at ORCI in Dar es Salaam, Tanzania. Using a purposeful sampling technique, 12 cervical cancer patients were interviewed with the principles of saturation guiding sample size determination. A semi-structured face-to-face interview guide was employed to collect the information. A conventional content analysis approach was used to analyze data after translation with the aid of NVivo 12.0 computer software.  Five themes emerged after data analysis: knowledge and attitude about cervical cancer, sufferings from a disease process, socio-economic disruptions, psychological problems, and sexual and reproductive concerns. |
| Introduction |  |  |  |  |
| Background/rationale | 2 | Explain the scientific background and rationale for the investigation being reported | 4-6 | Cervical cancer continues to be a major global public health concern and the leading cause of morbidity, mortality, and increased cost of treatment worldwide [1, 2]. In 2020, the estimated number of women who were diagnosed and died due to cervical cancer was 604,000 and 342,000 respectively, whereby about 90% were from low-and middle-income countries [3]. Africa accounts for 20% of the new global cervical cancer diagnoses annually, with about 120, 000 new cases [4]. Also, the burden of cervical cancer continues to be substantial in Sub-Saharan African (SSA) countries which contribute to 24.55% of global deaths [5]. Tanzania is among Sub-Saharan African countries with 4^th^ highest incidence rate of 59.1 new cases per 100,000 women in the world. The country has a high mortality rate of cervical cancer, with 42.7 deaths per 100,000 women [6]. The high burden of cervical cancer in LMICs is associated with limited access to public health services and poor implementation of screening, and treatment of this disease [4, 7, 8].  The World Health Organization (WHO) developed a global strategic initiative to eliminate the burden of cervical cancer by 2030. To achieve this goal, 90-70-90 ambitious targets have been set; 90% of girls should have received a full dose of the human papillomavirus (HPV) vaccine, 70% of women should have screened for cervical cancer twice, and 90% of women diagnosed with pre-cancer/cancer receive treatment and management; this is expected to eliminate a frequency of 4 per 100,000 women-years [3, 9]. The Ministry of Health in Tanzania has adopted the WHO global strategic initiative to develop an intervention plan to prevent, eliminate and manage cervical cancer cases. By 2024, Tanzania planned to have a target vaccination coverage rate and target screening coverage rate of 85% and 35% respectively [6]. The National Cervical Cancer Prevention and Control Strategic Plan with four elements of cervical cancer control and prevention was also introduced in the country. First, the prevention of risk factors that cause cervical cancer through the provision of health education for all people and encouraging the use Human Papillomavirus (HPV) Vaccine (primary prevention). Second, early detection of cancer targeting both girls and women through cervical cancer screening and treatment of precancers (secondary prevention). Third, the treatment of women who are diagnosed with cervical cancer (tertiary prevention). Fourth, the provision of palliative care to patients with cervical cancer in the advanced stage aimed to reduce pain, control bleeding, manage the side effects of drugs, and give comfort to the patients [10].  Lived experience with cervical cancer involves knowledge or experiences of a person with cervical cancer for those who are exposed to this medical condition [11]. Good living experiences with different medical conditions are associated with being knowledgeable about the causes, signs, symptoms, and treatments of the condition [12]. Patients with good living experiences with chronic illnesses are more likely to have good health-seeking behaviors and ultimately maintain their health statuses [13, 14]. Women with negative experiences with cervical cancer may use abnormal changes happening in their bodies to know that something is wrong with them [15]. Impacts of cervical cancer such as disruption of the patient physically, psychologically, socially, and economically enable us to know the experience of women who are diagnosed with cervical cancer [16]. Women who are diagnosed with cervical cancer may experience a reaction to diagnostic testing results, isolation from community and family members, marriage break-ups, anxiety, sadness, and deterioration of health status [14, 17]. Women diagnosed with cervical cancer experienced stigmatization from community members because most community members had misconceptions about cervical cancer [18, 19]. Other women with cervical cancer reported being physically isolated and not invited to various community events [20, 21].  The life-threatening experiences among women with cervical cancer have been further revealed in healthcare settings as they experience dissatisfaction with healthcare, inadequate information from healthcare providers, and difficulty tolerating treatment regimens [22, 23]. Studies conducted in Ghana and Uganda revealed that women with cervical cancer experienced Health deterioration caused by illness and treatment, psychological disturbance after receiving a positive diagnostic test for the disease, and experiencing symptoms of the disease have also been reported [15, 22]. Other women experience economic challenges (loss of working ability, loss of job, and high medical costs) and disruption of social relationships (marriage break-up, isolation by family and community members) [21, 24].  Despite the significance of understanding the lived experiences of cervical cancer patients in identifying the gap in health care among the affected population for better treatment and reduction of cervical cancer-related mortality, very few studies have been done so far, especially in Sub-Saharan African countries. This indicates a need to conduct more studies to get adequate information that will help key stakeholders in health care develop proper strategies to address this problem. |
| Objectives | 3 | State specific objectives, including any prespecified hypotheses | 6 | This study aimed to explore the lived experiences and caring needs of cervical cancer patients at Ocean Road Cancer Institute in Dar es Salaam, Tanzania. |
| Methods |  |  |  |  |
| Study design | 4 | Present key elements of study design early in the paper | 7 | A qualitative descriptive study design was employed to explore the lived experiences and caring needs of cervical cancer patients at ORCI in Dar es Salaam, Tanzania. This design was employed because it allows an in-depth exploration of participants’ experiences as proposed by Doyle *et al*., [25]. |
| Setting | 5 | Describe the setting, locations, and relevant dates, including periods of recruitment, exposure, follow-up, and data collection | 7 | This study was conducted at Ocean Road Cancer Institute (ORCI) in Dar es Salaam, Tanzania from December 2022 to January 2023. The ORCI is a national referral public center for cancer treatment and approximately 5,500 new patients with cancer are attended annually, of which approximately 39% have cervical cancer. The hospital is staffed by many specialized health professionals from several disciplines and receives all cancer patients referred from all regions in the country. The center provides chemotherapy, radiation therapy, complaint therapy, and other supportive and palliative care. It is the major area for cancer registry, early detection, prevention, standard treatment, and palliative care in Tanzania and it is the only cancer center in Dar es Salaam. |
| Participants | 6 | *Cross-sectional study*—Give the eligibility criteria, and the sources and methods of selection of participants | 7 | This study involved a population of women diagnosed with cervical cancer at ORCI in Dar es Salaam, Tanzania. All women with cervical cancer for at least 1 year since diagnosis at the Institute were included in this study. Critically ill cervical cancer patients were excluded from this study because they could not be comfortable in responding to the questions due to potential life-threatening physiological conditions requiring critical care. Also, all cervical cancer patients who had cognitive impairment were excluded from this study because they could not provide the required information. |
| Bias | 9 | Describe any efforts to address potential sources of bias | 9-10 | To ensure the credibility of the findings, the authors discussed meanings emerging from the analysis outputs, categories, and themes according to the specific objectives of this study. The authors also held a meeting with interviewed participants to discuss and reach a consensus on the themes and subthemes identified. Their suggestions were obtained regarding the comprehensiveness of the findings and changes were incorporated accordingly. |
| Study size | 10 | Explain how the study size was arrived at | 8 | A purposeful sampling technique was used to recruit the study participants. Patients’ records of patients were reviewed at the registry point to identify those who had been diagnosed with the disease. Cervical cancer patients who were able and willing to provide descriptions of their experiences and needs with the disease were purposively selected from those who met the inclusion criteria. A total of 12 participants were interviewed with sample size determination based on the principles of saturation as proposed by Malterud *et al.,* [26], i.e.; sampling was terminated when no new information was obtained from the study participants. |

Continued on next page

| Statistical methods | 12 | (*a*) Describe all statistical methods, including those used to control for confounding | 9-10 | Data analysis was initiated soon after the first interview by transcription of the audio-recorded data by typing directly into the computer with the aid of the Microsoft Word program and then translating into the English language. Iterative reading of the transcripts was done by Principal Investigators separately with an open mind to obtain a general impression and an overall understanding of each transcript. Different colors were used to highlight the patterns in the text corresponding to the preconceived category stated in the study objectives. The transcribed and translated data sets were transferred to NVivo 12.0 computer software which is designed to help in organizing the qualitative data and coding the text. The conventional content analysis approach was employed to enable a deeper understanding and formation of themes after several reading iterations of the transcripts as proposed by Hsieh & Shannon [27]. Categories were developed from actual phrases in the text segments and sub-themes were extracted from each category by the Principal Investigators. Similar categories were linked to form themes (***Fig 1***). Field notes were also analyzed separately whereby the patterns and categories were compared to those from in-depth interviews. Data in the form of direct quotes were used to substantiate the relevant categories. To ensure the credibility of the findings, the authors discussed meanings emerging from the analysis outputs, categories, and themes according to the specific objectives of this study. The authors also held a meeting with interviewed participants to discuss and reach a consensus on the themes and subthemes identified. Their suggestions were obtained regarding the comprehensiveness of the findings and changes were incorporated accordingly. |
| --- | --- | --- | --- | --- |
| Results |  |  |  |  |
| Participants | 13* | (a) Report numbers of individuals at each stage of study—eg numbers potentially eligible, examined for eligibility, confirmed eligible, included in the study, completing follow-up, and analysed | 8,11 | A total of 12 participants were interviewed with sample size determination based on the principles of saturation as proposed by Malterud *et al.,* [26], i.e.; sampling was terminated when no new information was obtained from the study participants. |
| Descriptive data | 14* | (a) Give characteristics of study participants (eg demographic, clinical, social) and information on exposures and potential confounders | 11 | A total of twelve participants participated in this study. The mean (±SD) age of participants was 53.3 (±14.5) years with half of the participants in the age group of 31 to 50 years. At the time of the interview, 9 participants had two years since being diagnosed with cervical cancer. Four participants were single, the other 4 were divorced, and 10 were Christians. Most (10 participants) had primary education and only one participant was formally employed. Half of the participants had a parity of 1 to 2 children and 9 had a stage II cancer diagnosis. Ten participants received concurrent radiotherapy and chemotherapy treatments. All participants had curative treatment intentions. |
| Outcome data | 15* | *Cross-sectional study—*Report numbers of outcome events or summary measures | 13 | Five predominant themes emerged after data analysis as follows: knowledge and attitude about cervical cancer, sufferings from a disease process, socio-economic disruptions, psychological problems, and sexual and reproductive concerns. |

Continued on next page

| Other analyses | 17 | Report other analyses done—eg analyses of subgroups and interactions, and sensitivity analyses | 13 | Five predominant themes emerged after data analysis as follows: knowledge and attitude about cervical cancer, sufferings from a disease process, socio-economic disruptions, psychological problems, and sexual and reproductive concerns. |
| --- | --- | --- | --- | --- |
| Discussion |  |  |  |  |
| Key results | 18 | Summarise key results with reference to study objectives | 25 | The present study explored the lived experiences and caring needs of women impacted by cervical cancer at ORCI in Dar es Salaam, Tanzania. The mere fact that women diagnosed with cervical cancer in the current study did not have palliative treatment intentions did not mean they were immune to devastating life experiences imposed by the disease. Being women diagnosed with cervical cancer in a resource-limited setting and having a low community understanding of the disease would not be so kind and favorable to them. Hence, the findings of this study revealed various biological, psychological, social, and economic challenges that cervical cancer patients experienced from the time they were diagnosed with the disease and started to manifest symptoms of the disease. Five predominant themes emerged after data analysis as follows: knowledge and attitude about cervical cancer, sufferings from a disease process, socio-economic disruptions, psychological problems, and sexual and reproductive concerns. |
| Limitations | 19 | Discuss limitations of the study, taking into account sources of potential bias or imprecision. Discuss both direction and magnitude of any potential bias | 29 | The limitation of this study is that all participants recruited had curative treatment intention, and there was no participant in the terminal stages of cancer. Therefore, the findings of this study might not reflect the lived experiences and caring needs of patients with terminal cervical cancer. |
| Interpretation | 20 | Give a cautious overall interpretation of results considering objectives, limitations, multiplicity of analyses, results from similar studies, and other relevant evidence | 25-28 | The findings of this study revealed the low level of knowledge about cervical cancer in terms of what causes the disease, risk factors, signs and symptoms, and treatment options available among patients themselves and society in general. The presence of knowledge gaps concerning cervical cancer compounded the negative attitudes towards the disease which was very detrimental to the overall psychosocial well-being of the affected population. The low level of knowledge and unfavorable attitude regarding cervical cancer has been elucidated by several other studies [14, 15, 17]. Such findings also distort the screening programs among women, leading to late disease detection and consequently late consultation for orthodox health services. These findings illuminate the need for responsible authorities in developing and delivering mass education programs concerning cervical cancer with much emphasis on risk factors, signs and symptoms, and treatment modalities available to enhance early screening and rule out prevailing poor beliefs regarding the disease [28, 29].  Participants from this study reported experiencing suffering attributed to news about the diagnosis, disease process, and treatment-related effects. The fact that most participants perceived cervical cancer as a death sentence, it was difficult for them to handle the news as a result they manifested maladaptive coping strategies like self-isolation. The news of the diagnosis affected other family members as they grieved for the impending death of their beloved ones. Consistent findings were reported from a study conducted in Japan among cervical cancer patients with terminal illnesses as they expressed experiencing extreme suffering and desired to at least die peacefully [19]. Also, this finding corroborates those of studies conducted among cervical cancer patients in Ghana and Zambia [14, 15]. Despite the participants from those studies suffering from the news of the diagnosis, the extent of suffering was quite different depending on the context of the study as settings with adequate knowledge of cervical cancer had minimal suffering because women knew the story behind the disease. In the current study, participants reported suffering from the physical symptoms of the disease and treatment side effects. Consistent findings were reported by adolescent cancer patients in studies conducted in Taiwan and Singapore [18, 30]. The most voiced symptom was bleeding that made women uncomfortable and some of them developed hematological deficits and nausea and vomiting were reported as debilitating for patients undertaking chemotherapy. More studies are needed to explore how better healthcare can be provided to relieve the sufferings associated with the symptom and ultimately promote healthier functioning for patients impacted by cervical cancer in such resource-limited settings [31].  The psychological problems experienced by cervical cancer patients in this study are similar to what was found in an exploratory study conducted in Ghana among cervical cancer patients where participants reported facing stressful experiences and depression after being frustrated with abusive interactions with some family members and society at large [15]. They were emotionally humiliated and psychologically tortured by cancer-related stigma due to the prevailing negative attitude towards cervical cancer in the community. Similar psychological distress was reported in a study done in Ethiopia among cervical cancer patients during follow-up care [22]. Hopelessness and lack of emotional support reported in the current study have also been reported by other studies done in different settings [14, 30, 32] where cancer was perceived as a death sentence for diagnosed patients. Public awareness programs are required to address such information gaps and rule out prevailing misconceptions concerning cervical cancer and other cancerous diseases in general [12]. Also, providing psychological and emotional support to women with cervical cancer as well as their caregivers is imperative for enhancing their capability among them to cope with the situation and lead a quality life. One of the ways to make this possible is by having cumulative efforts among healthcare providers and allied stakeholders in developing tangible strategies to address the concern [28].  Women with cervical cancer experienced disruptions in social and economic aspects of their life, which could be explained by a reduced capability to produce as they used to do and social isolation due to limited understanding and negative attitudes concerning cervical cancer. Also, the context itself from where patients were living was resource-limited by nature and thus the ability to acquire basic needs was dependent on their daily activities. So, having cervical cancer with such debilitating physical symptoms reduced their abilities to perform usual productive activities leading to an economic crisis among them and their families. The cost of treatments also rendered some patients’ families bankrupt with others forced to sell the assets they had to cater for such costs. Similar findings of socioeconomic disruptions as a result of the disease were reported in other studies [14, 15, 22]. It was also revealed that the disruption imposed by cervical cancer on social and economic aspects of patients’ life was a source of constant stress, low self-esteem, and isolation [14, 33]. This finding in the current study implies that healthcare providers should conduct a regular individualized assessment to explore patients’ social and financial needs to ascertain the extent of socio-economic disruptions for early and proper linkage to social support groups [34].  The findings of this study also revealed the presence of sexual and reproductive concerns among women with cervical cancer. Husbands of women diagnosed with cervical cancer were reported to isolate their sick wives leading to family breakouts. Lack of knowledge about the disease among men geared up their decisions to abandon their wives as they perceived cervical cancer to have a detrimental effect on women’s reproductive health by making them no longer fertile. Having such sexual and reproductive concerns is not limited to Tanzanian women in this study but is also revealed by women with cervical cancer in studies conducted in other Sub-Saharan African countries [14, 22]. A study conducted in Uganda among patients diagnosed with cervical cancer revealed that women experienced disruption in their sexual relationships including being deserted by their partners and family [17]. Women with cervical cancer revealed that they were scared of the unknown about their fertility particularly after undergoing radiotherapy and chemotherapy treatments. This may have negative psychological implications leading to a delayed recovery process and maladaptive coping behaviors among the affected women [35]. Comprehensive and holistic care should be given to women and their families in general to tackle the unmet needs among couples concerning sexual and reproductive concerns in relation to the disease itself, treatment modalities, and ultimate prognosis [36, 37]. However, other factors related to the sexual and reproductive health of women impacted by cervical cancer need further investigation. |
| Generalisability | 21 | Discuss the generalisability (external validity) of the study results | N/A | The findings of the present study are based on the lived experiences and caring needs of women diagnosed with cervical cancer at Ocean Road Cancer Institute in Dar es Salaam, Tanzania. Therefore, it may have affected the generalizability of this study’s findings to other populations in other settings. |
| Other information |  |  |  |  |
| Funding | 22 | Give the source of funding and the role of the funders for the present study and, if applicable, for the original study on which the present article is based | N/A | The authors received no financial support for the authorship and/ or publication of this study. |
