# Supplementary material for: The lived experiences and caring needs of women diagnosed with cervical cancer: A qualitative study in Dar es Salaam, Tanzania
Source: PLoS One. 2023 Aug 10;18(8):e0289925. doi: 10.1371/journal.pone.0289925 (PMC10414621; doi:10.1371/journal.pone.0289925)
Supplement: S3 Text — (DOCX) [file pone.0289925.s003.docx]

# **The lived experiences and caring needs of women diagnosed with cervical cancer: A qualitative study in Dar es Salaam, Tanzania**

| Table 1. Socio-demographic characteristics of study participants | |
| --- | --- |
| Variables | **Frequencies (n)** |
| Age in years  31-50  51-70  71-90 | 6  3  3 |
| Duration of cervical cancer since diagnosis  One year  Two years  Three years and above | 2  9  1 |
| Marital status  Single  Married  Widow/widower  Divorced | 4  2  2  4 |
| Religion  Christian  Muslim | 10  2 |
| Level of education  No formal education  Primary education | 2  10 |
| Occupation  Formally employed  Self-employed  Unemployed | 1  8  3 |
| Parity  0  1-2  3-5  6 and above | 1  6  2  3 |
| Current stage of cancer  Stage I  Stage II | 3  9 |
| Treatment intent  Curative  Palliative | 12  0 |
| Radiotherapy concurrent with chemotherapy  Yes  No | 10  2 |

**Exerpts of the transcipts**

**Knowledge and attitude about cervical cancer**

*“No, there is no one there who understands that cancer can be cured, that is, for someone who has not been here. I do not believe that there is someone in the street or the village who can know if cancer is curable, because if you tell someone that you have cancer, he/she gets a big shock”* (Participant 2).

*“About cancer treatment, they know it doesn’t exist, for instance, there was my nephew who was suffering from breast cancer, and she kept on injecting herself with painkillers. It has been too long now since she was admitted here and she died. Therefore, the family in general still believes that the chemotherapy and radiation therapy she was initiated here is what killed her”* (Participant 10).

*“I don’t know why you men are killers like this, can you imagine I have been faithful to him for more than 4 years now but he didn’t care. He cheated me and came back with this deadly disease. I can’t trust a man anymore after all these tragedies”* (Participant 6).

*“There were changes, we were sad because they were built with the belief that cancer is incurable, so my mother and my children were very sad. Some of my other brothers were among the first people to disappoint me as they were saying and announcing that cancer is incurable. Moreover, they claimed that here at ORCI where I came for treatments, many people die, thus they believed I won’t get back home alive also”* (Participant 1).

*“Also, other brothers think that this disease is not healing, that is, they think why should we give our money to someone who has already been infected with this disease. They do not believe that this treatment is curative, so they don’t believe I will recover”* (Participant 10).

**Sufferings from a disease process**

*“I received the news that I was diagnosed with cervical cancer very badly and I was very sad. I was overwhelmed with thoughts until I lost consciousness. With such suffering I was experiencing, my family including my mother also disappointed me after revealing the diagnosis, they were saying that cancer can’t be cured*” (Participant 7).

*“I was very affected because I was unable to eat after experiencing this problem. I completely lost my energy even to do other work, I was unable to do it at that time as my body was completely lacking strength”* (Participant 11).

*“...that is, when I was diagnosed, I went to tell my family that I had been diagnosed with cervical cancer, they were all confused and some believed that I was on my way to die”* (Participant 5).

“*When I told my mother about the news, she was sad and she stayed like this for a week, she doesn't drink and she left the house, I went out to search her and fortunately I caught her and urged her to return home. She started telling me “My soul is hurting my daughter, you are dying*” (Participant 12).

*“I was very shocked when I was at home because the blood was coming out too much, I was a bedridden person and the blood was not cutting at all. Until I came here, I’m thankful, I have done the treatments and nowadays the blood comes out a little”* (Participant 9).

*“I was surprised myself; the blood was coming out profusely and that is why I even told my brothers that I don’t know whether I will recover from the disease. It was hurting me a lot, that’s why I thought it would be better to come here and start the treatments”* (Participant 12).

*“When I started the radiation, it caused me nausea, it was causing problems. There were days when I couldn't eat, that is, I didn't want any kind of food, so I stayed until the evening without eating anything”* (Participant 3).

*“The negative side effects of the treatment for me are like nausea when I was taking chemotherapy, I used to lose energy and I felt headaches too”* (Participant 8).

**Socio-economic disruptions**

*“My personal life has been affected, when I was growing up I was looking for a job to earn an income. Right now, I'm stuck because I'm sick. Also, my child's life has been affected because my child does not have a job, he always does people's work. Up to now, my grandchildren have not gone to school since the school opened. He is struggling to find the money for treatment and his children. The grandchildren have been suspended from school because they have no shoes, they don't have the things needed at school*” (Participant 9).

“*The economy was the most unstable even now as I speak, the situation is not good because I can no longer continue with my activities of selling food that I was doing and earning a little income, I was supporting my children and my mother (considering that my mother is disabled). When I left the house to come here for treatment, I left my mother and my children without any help*” (Participant 1).

*“In terms of the economy, there is a lot that has affected me because the situation I had made me unable to do my work with such success I used to get. In the evening I usually used to prepare porridge and send it to sell but when it reached this point, my customers were annoyed with the situation I was passing through as blood sometimes came out profusely and customers disliked my porridge as I was dirty. It means that I was unable to stay in the crowd and do those jobs. I was staying with my children who are students and were by then missing their basic needs*” (Participant 2).

*“After having this problem, my sister ran away from me, and until now I have no information about her. Before I shared this information about the disease, our relationship was good, but after I shared it with her, she became useless to me and until now I don't know where she is and even communication became a problem”* (Participant 3).

*“My brothers have not been involved with my illness because they were against my decision to come here for treatment. They know that the drugs here are very expensive but they don’t give any support just because I went against their wishes of not coming for treatments here. They wanted to preserve the family resources as they believed the disease can’t be cured and thus, they better not spend money catering for palliative care”* (Participant 4).

**Psychological problems**

*“I feel bad, very bad because they don’t support me in this situation…that is, out of ten people in the family, only three are in contact with me…Now I don't know, maybe due to the difficulty of life, that's why they don't give me help, I don't know actually and I don't understand what is behind this tragedy, because others have good financial abilities, but I don't understand why they don’t support me”* (Participant 3).

*“My eldest son who is 29 years old has also joined that group of my brothers, he does not show concern or give me any cooperation, not even a phone greeting. This has affected me greatly psychologically because I used to think that I am a human being just like them, but due to these tragedies that have happened to me, it has made them see me as if I’m not their fellow human being and family member”* (Participant 1).

*“Before I shift the settlement to here at the hospital, I used to attend the clinic from my relatives here in the city. They discriminated against me a lot to the extent I had depression, for instance, when I went for a short call in the toilets, they immediately visited the place and cleaned it. They thought that I will infect them with the disease if we could share such facilities at home. Even food they saved mine separately as they believed the disease was contagious”* (Participant 10).

*“When they went to test me, the soldier started to shout in front of me and said aaah aisee! Afande…this mother has nothing at all, her cervix is all eaten up and also has active bleeding. He further insisted that nothing can be done to save my life and thus I have to return home”* (Participant 11).

**Sexual and reproductive concerns**

*“Normal life is that, after I had this situation, my husband did not enjoy the situation after I met him, blood spilled out and through that, it contributed to the separation from my husband. He didn't know if I had cancer, but when the symptoms started, he became disinterested in it and as I’m talking now, he ran away from me and has another woman there”* (Participant 2).

*“When my partner saw that I was sick like this, he just left me because he believed I would not recover and satisfy his sexual desires as before. Being abused because of my illness I think was the reason for our family separation as he used to snub my concerns at the moment. He already assumed that I will not be able to participate in sexual intercourse with him”* (Participant 5).

*“I can say that my husband is a colonist because after I told him about my limited comfortability with sexual intercourse, he has taken everything from me. So, I am left with nothing. Even when I came here, he didn't give me anything, therefore, I couldn't even buy drinking water on the way”* (Participant 6).

*“We are harassed a lot by men once we have this disease because they run away from home with the assets we had as a family. For instance, he told me that when I came back, I will stay with my children and I won’t find him as he will get married to another healthy lady”* (Participant 7).

*“I stopped sharing with my husband because I was really scared. I told him that it was for the sake of my life rather than giving birth later on, so I only did a little with him. He is a colonist, he doesn’t realize that this person is sick, so sometimes I run away from him because I consider myself weak and no longer fertile”* (Participant 8).

*“Because right after I started the treatments here, I had a lot of problems, that is, I asked the doctor if I will conceive again amid all the radiation I was getting. It means that this problem that happened just came as another emergency but I didn't think about it too much because I had three children already”* (Participant 4).
