# Supplementary material for: The lived experiences and caring needs of women diagnosed with cervical cancer: A qualitative study in Dar es Salaam, Tanzania
Source: PLoS One. 2023 Aug 10;18(8):e0289925. doi: 10.1371/journal.pone.0289925 (PMC10414621; doi:10.1371/journal.pone.0289925)
Supplement: Attachment — Submitted filename: Response to Reviewers.pdf [file pone.0289925.s004.pdf]

July 13, 2023

To

Editor-in-Chief

PLOS ONE Journal

Dear Sir/Madam,

On behalf of my co-authors, I am grateful for the valuable and constructive review of our manuscript **PONE-D-23-10682** entitled **“The lived experiences and caring needs of women diagnosed with cervical cancer: A qualitative study in Dar es Salaam, Tanzania”**. We have revised the manuscript according to editor and reviewers’ comments and made clarifications where required. The specific response to editor and reviewers’ comments is elaborated in details within the matrix response sheet below after this letter.

Yours Sincerely,

………………

Emmanuel Z. Chona

Email: emmanuelchona20@gmail.com

Matrix response sheet on academic editor and reviewers' comments to manuscript PONE-D-23-10682 entitled "*The lived experiences and caring needs of women diagnosed with cervical cancer: A qualitative study in Dar es Salaam, Tanzania*"

**Editor's comments**	**Response from author**
It should be noted in the methods that all study participants had early-stage disease	We have added in the methods that 12 early-stage cervical cancer patients were recruited for the study.<br>Please see the revised manuscript (Line 125).
The background should be made more concise	The background has been revised to make it more concise.<br>Please see the revised manuscript.
There is redundancy in the quotations, please remove redundant passages	We have revised the analysis critically and we removed redundant quotations reporting the same category in a specific theme.<br>Please see the revised manuscript.
The English needs polishing	The revised manuscript has been thoroughly copyedited by an expert university faculty competent in English with a strong scientific background in the field of medical research to make it clear and unambiguous.<br>Please see the revised manuscript.

**Reviewer's comments**	**Response from author**
Is the manuscript technically sound, and do the data support the conclusions?<br>The manuscript must describe a technically sound piece of scientific research with data that supports the conclusions. Experiments must have been conducted rigorously, with appropriate controls, replication, and sample sizes. The conclusions must be drawn appropriately based on the data presented.	After we reviewed all parts of our manuscripts followed the reviewer's comments, we found some technical errors of our manuscript and we corrected them accordingly. We ensured that the conclusions made in the manuscript are drawn appropriately based on our study findings presented.<br>Please see the revised manuscript.
Have the authors made all data underlying the findings in their manuscript fully available?	After we reviewed the PLOS Data policy, we have agreed to share our minimal anonymized data set (Excerpts of transcripts) as supporting information.
Is the manuscript presented in an intelligible fashion and written in standard English?	The revised manuscript has been thoroughly copyedited by an expert university faculty competent in English with a strong scientific background in the field of medical research to make it clear and unambiguous.<br>Please see the revised manuscript.
The authors quite rightly note that a limitation was not interviewing women with cervical cancer who have progressed and maybe dying.	We have revised the manuscript to incorporate the recommendation that further qualitative studies should be done to explore the lived

So, the authors should add in their discussion that future studies should include this group.	experiences and caring needs of terminal cervical cancer patients.<br>Please see the revised manuscript (Line 503-506).
In the data analysis (line 175) it is specified that some statements in the transcribed interviews were highlighted according to the preconceived categories stated in the study objectives, which is not the case, please clarify or report them	During the analysis process, authors performed iterative reading of the transcribed data to obtain a general impression and an overall understanding of each transcript. With the objective of this study being exploration of the lived experiences and caring needs of cervical cancer patients, hence meaning units reflecting the study's objectives were highlighted with different colors during iterations for easy generation of patterns and codes from the text. Hence, categories were developed from actual phrases in the text segments and similar ones were linked to generate themes.<br>Please see the revised manuscript (Line 151-160).
Question 7 exploring the experience with health care services are not mentioned and it is not part of the themes and categories, explain why	The experience with health care services were explored as per question 7. During the analysis process, several codes emerged concerning the health care services which were used to

	generate different categories reported in the manuscript. For instance, high costs associated with radiation therapy, high consultation fees, limited capability to purchase chemotherapy drugs were among the codes revealed by some participants under the aspect of health care services which were then linked with other codes arising from response to different questions like reduced capability to perform productive activities to generate a category of financial hardship. Also, codes like chemotherapy associated side-effects (example nausea and vomiting) were revealed upon probes on health care services which were then combined with other codes like radiotherapy associated side-effects to generate a category of treatment side-effects. Please see the revised manuscript.
Line 184, to ensure credibility. Credibility is not a term used in qualitative analysis, do the authors mean reliability? Please clarify	By stating credibility, we meant the degree to which the results of a qualitative research are credible or believable from the participant's perspectives since the purpose of qualitative is to describe or understand phenomena of

	interest from participants, hence they are the only ones who can legitimately judge the trustworthiness of the results. With this technique of establishing trustworthiness, data interpretations and conclusions were discussed by investigators and shared with the participants to allow them to clarify what their intentions were, correct misunderstandings and provide additional information if necessary.
The quotes taken from the interviews represent a significant part of the paper, there are overlapping and they make difficult the reading. I suggest to reduce their number, to keep the significant statements and report them in extent with references to the paper in the appendix	We have revised the analysis critically and we removed redundant quotations reporting the same category in a specific theme.<br>Please see the revised manuscript.
English to be fully and carefully revised	The revised manuscript has been thoroughly copyedited by an expert university faculty competent in English with a strong scientific background in the field of medical research to make it clear and unambiguous.<br>Please see the revised manuscript.
